# No Evidence for Effect of Exercise on Transcriptome of NK Cells in Breast Cancer Patients Undergoing Adjuvant Therapy: Results From a Pilot Study

**DOI:** 10.3389/fphys.2019.00959

**Published:** 2019-07-25

**Authors:** Anasua Pal, Philipp Zimmer, Martina E. Schmidt, Manuela Hummel, Cornelia M. Ulrich, Joachim Wiskemann, Karen Steindorf

**Affiliations:** ^1^Division of Physical Activity, Prevention and Cancer, German Cancer Research Center (DKFZ), Heidelberg, Germany; ^2^National Center for Tumor Diseases, Heidelberg, Germany; ^3^Department of Molecular and Cellular Sport Medicine, Institute of Cardiovascular Research and Sports Medicine, German Sport University Cologne, Köln, Germany; ^4^Division of Biostatistics, German Cancer Research Center (DKFZ), Heidelberg, Germany; ^5^Department of Population Health Sciences, Huntsman Cancer Institute, University of Utah, Salt Lake City, UT, United States; ^6^Division of Medical Oncology, Heidelberg University Hospital and National Center for Tumor Diseases, Heidelberg, Germany

**Keywords:** exercise, physical activity, cancer, NK cell, transcriptome, gene expression

## Abstract

**Background:**

Mobilization and activation of natural killer cells (NK cells) have been hypothesized to contribute to observed protective effects of exercise on cancer development and progression. Some evidence exists for acute effects of aerobic exercise on NK cell mobilization and function, i.e., alteration of the gene expression profile of NK cells. Yet, the chronic effects of exercise training, and effects of other modalities than endurance exercise are still understudied. Here, we investigated the chronic effects of a 12-week resistance exercise program on NK cell gene expression in breast cancer patients undergoing adjuvant chemo- or radiotherapy.

**Methods:**

Breast cancer patients were randomly assigned to either a 12-week resistance exercise program or a relaxation control group concomitant to adjuvant therapy. In a subsample of 19 participants, RNA was extracted from magnet bead isolated NK cells and subsequently analyzed for differential gene expression using microarray Illumina HumanHT-12 v4 before and after the intervention.

**Results:**

After chronic exercise intervention several genes showed higher differential expression compared to the control group. However, after correction for multiple testing, baseline-adjusted analyses of covariance indicated no significant differences between the intervention and the control group with regard to the gene expression profile.

**Discussion:**

Our findings suggest that 12-week resistance-exercise did not alter the gene expression profile of NK cells in breast cancer patients undergoing adjuvant therapy on the long term. Further studies with larger sample sizes and specifically designed to investigate whether exercise-induced changes in NK cell function are attributed to acute effects are warranted.

## Introduction

An active lifestyle is associated with reduced breast cancer risk and improved survival in breast cancer patients (Bodai and Tuso, 2015). Besides a reduction of chronic inflammation, physical exercise has also been shown to regulate hormonal balance including catecholamine, prostaglandins, and cortisol (Keast et al., 1988). Additionally, exercise is known to influence cytokine levels and number, proportions and functions of various immune cells which are involved in the host tumor defense (Lancaster, 2006). One of the most frequently investigated immune cell populations in the context of exercise and cancer are natural killer cells (NK cells) (Pedersen et al., 2016). As a part of the innate immune system NK cells have the potential to identify and eliminate tumor cells without prior priming. Activity of NK cells is dependent on the interplay between its activating and inhibiting receptors. Indeed, higher levels of intra-tumoral NK cell numbers are associated with better prognosis in some types of cancer (Larsen et al., 2014).

Studies have revealed that an acute bout of exercise affect the NK cell distribution and function. In detail, NK cells are elevated immediately after exercise, strongly decrease in the subsequent hours and return to baseline within 24 h (Nieman, 1997), depending on exercise duration and intensity. In view of NK cell function, various studies reported elevated NK cell cytotoxicity levels after a single bout of exercise. On a more mechanistic level, Radom-Aizik et al. (2013) reported acute effects of a brief bout of exercise in 12 participants on the gene and miRNA expression pattern in NK cells. Their findings reveal seven pathways that are related to cancer and cell communication and changed the expression level of 23 miRNAs. The seven pathways are possibly regulated by miRNAs. Activation of NK cells is dependent on the interplay between its activating and inhibiting receptors. Additionally, their results also found upregulation of three KIR activation receptors, KIR2DL4, KIR2DS3, and KIR2DS4, in the circulating NK population in response to acute exercise. Another recent study has found that acute exercise leads to global histone modifications in NK cells and a subsequent increase in the expression of the activating NK cell receptor NKG2D (Zimmer et al., 2015).

In contrast to acute effects of exercise on NK cell mobilization and function, the knowledge about training effects on baseline NK cell function is still sparse. Although inconsistent, some studies reported changes of resting NK cell numbers and proportions as well as altered NK cell cytotoxicity after longer training periods (Gleeson et al., 1995).

Since acute effects of exercise on NK cell cytotoxicity are accompanied by changes of the transcriptome we hypothesized that chronic physical exercise may have a similar effect. Thus, in this pilot study we investigated the chronic effect of 12-week resistance exercise on NK-cell gene expression in breast cancer patients undergoing chemotherapy or radiation therapy.

## Materials and Methods

### Research Design

The BEST and BEATE studies were randomized, controlled intervention trials investigating the effects of resistance exercise training in 261 stage 0–III breast cancer patients during adjuvant radiation therapy or adjuvant chemotherapy, respectively. Before the start of intervention (baseline, T0), and after the end of intervention (T2), endpoints and patients characteristics were assessed and blood samples taken. The studies were approved by the ethics committee of the University of Heidelberg and registered at ClinicalTrials.gov (NCT01468766, NCT01106820). Details of the study designs and primary results are already published (Potthoff et al., 2013; Schmidt et al., 2013, 2014; Steindorf et al., 2014; Liu et al., 2017). Here we analyzed a homogenous subgroup of participants from these studies in order to determine the physiological adaptions of 12-week resistance training.

### Participants

The consent obtained from all participants was both informed and written. General exclusion criteria for the exercise trials comprised contra-indications for progressive resistance training and patients performing already a systematic intense resistance training (at least twice a week) were excluded. In order to investigate a homogenous sample, 10 participants of each trial (BEATE and BEST) were carefully selected for immunological analysis. Participants were chosen by indicating similar baseline values of BMI, age, and fitness levels between both trials (BEATE and BEST) as well as between participants of the intervention and the control groups. Participants of the exercise groups were only considered for inclusion if they attended >80% of training sessions. Moreover, participants who reported acute infections prior or during blood sampling were excluded.

### Exercise Protocol

Both interventions were performed group-based for 60 min twice weekly over 12 weeks under the supervision and guidance of experienced therapists in specific training facilities. Exercise comprised eight different machine-based progressive resistance exercises (three sets, 8–12 repetitions at 60–80% of one repetition maximum) without any specific aerobic exercise. The control group encompassed progressive muscle relaxation according to Jacobson without any aerobic or muscle strengthening exercise (Schmidt et al., 2013).

### NK Cell Isolation

PBMCs were isolated from freshly collected blood. After PBMC isolation with Ficoll, the cells were adjusted to a maximum of 5 ^*^ 10^7^ cells/ml and were re-suspended in 1 l phosphate buffer solution (PBS). NK cells were isolated from peripheral blood mononuclear cells (PBMCs) using EasySep Human NK-Cell Enrichment Kit, StemCell Technologies (Cat# 19055).

### RNA Extraction

Total RNA for gene and miRNA analysis was extracted using TRIzol (Ambion by Life Technologies). For gene expression study only, RNA was purified using Qiagen-RNeasy Mini Kit. RNA pellets were re-suspended in diethyl pyrocarbonate-treated water. RNA integrity was assessed (before beginning target processing) using Agilent Bioanalyzer 2100 (Agilent Technologies, Palo Alto, CA, United States).

### Gene Expression Microarrays

Microarray analysis using Illumina HumanHT-12 v4 Expression BeadChip was conducted with the extracted RNA of NK cells. Each array on the HumanHT-12 v4 Expression BeadChip targets >47,000 probes. A microarray is used to detect the expression levels of thousands of genes at the same time. BeadChips microarray consists of oligonucleotides immobilized to beads held in microwells on the surface of an array substrate. RNA collected from isolated NK cells was labeled using the TargetAmp-Nano^TM^ Labeling Kit. Labeled RNA was detected by hybridization to the probes on the BeadChip. After washing and staining steps, BeadChips are scanned on a iScan^TM^. The experiment was carried out in adherence to the protocol provided by Illumina Technologies^®^.

### Data Analysis

Microarray scanning was done using an iScan^TM^ array scanner. Data extraction was done for all beads individually, and outliers were removed when the absolute difference to the median was >2.5 times median absolute deviation (MAD) (2.5 Hampel’s method). All remaining bead level data points were then background corrected and quantile normalized (Shi et al., 2010). As test for significance the student’s *t*-test was used on the (log2 scaled) bead expression values of the two groups of interest. In the case of significance of expression against background we tested for greater than all negative beads for this sample and in the case of comparing separate groups we tested for inequality of the means of the groups. In the case of comparing groups we additionally calculated *p*-values using averaged expression values for each sample in the group. For the differential expression analysis, Benjamini–Hochberg correction was applied to the complete set of *p*-values of all ProbeIDs on the chip. The average expression value was calculated as mean of the measured expressions of beads together with the standard deviation of the beads. The test for baseline-adjusted differences between exercise vs. non-exercise after oncological treatment was done using analysis of covariance (ANCOVA) expression after treatment as dependent variable and expression at baseline as well as medical treatment as covariate. Testing several thousands of genes individually requires adjustment for multiple testing in order to control for false-positive findings. We adjust *p*-values using the Benjamini–Hochberg method, which controls the false discovery rate (Benjamini and Hochberg, 1995). All statistical analyses were performed using the R statistical environment.

## Results

Transcriptome analysis from one participant of Chemotherapy intervention group (BEATE) was not successful; hence, the total sample size is 19. Participant’s characteristics are shown in Table 1.

**TABLE 1:** Participant characteristics.

		**Total**		**Exercise**		**Control**	
Total, *n* (%)		19	100%	10	100%	9	100%
Age, mean (*SD*) (years)		51.1	(5.8)	51.8	(7.8)	50.2	(1.9)
BMI, mean (*SD*) (kg/m^2^)		23.8	(3.3)	24.5	(4.1)	22.9	(2.0)
VO2peak (ml/min), mean (*SD*)		1570	(347.0)	1615	(405.5)	1522	(282.7)
Treatments, *n* (%)	Chemo therapy	9	45.5%	6	66.7%	3	33.3%
	Radiation therapy	10	54.5%	4	40%	6	60%
Stage, *n* (%)	0	1	4.5%	1	8.3%	0	0%
	1	10	45.5%	3	25.0%	7	70.0%
	2	8	36.4%	6	50.0%	2	20.0%
	3	3	13.6%	2	16.7%	1	10.%

**n*, number of participants; SD, standard deviation.*

**FIGURE 1:**
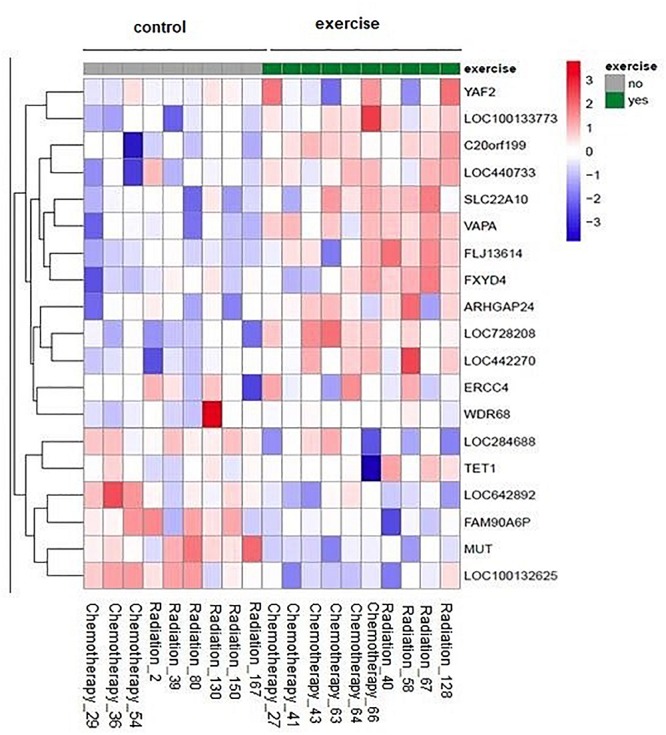
Impact of physical exercise on NK cell gene expression: The heat map represents the scaled expression levels of genes with raw *p* < 0.001. The rows represent genes identified by the name on the right of the figure. The individual patient samples are shown as columns (1 column per sample). The color red represents high expression, while blue represents low expression. The heat map is combined with clustering where genes are grouped together based on the similarity of their gene expression pattern.

There were no significant group × time interactions after correcting for multiple testing (all adjusted *p*-values > 0.9). However, we could explore the top candidates, e.g., the attached heat map contains the genes with raw *p* < 0.001 (Figure 1).

One of the interesting candidates is the TET1 gene (10–11 translocation methyl cytosine dioxygenase 1). The expression levels after the treatment tend to be higher in the exercise group compared to the non-exercise group (raw *p* = 0.0008, adjusted *p* = 0.990912).

FXYD4 is an ion transport regulator gene expression and has a significant difference in expression between exercise and non-exercise groups with a higher expression in exercise group (raw *p* = 0.0003, adjusted *p* = 0.990912).

The expression of VAPA, *Homo sapiens* vesicle-associated membrane protein (VAMP)-associated protein A was higher in exercise group compared to the non-exercise group after treatment (raw *p* = 0.00007, adjusted *p* = 0.990912).

The expression levels of *Homo sapiens* WD repeat domain 68 (WDR68) after treatment tend to be higher in the exercise group compared to the non-exercise group. This effect was still visible when analysis was performed without the “outlier” sample in the non-exercise group with very low expression before treatment (raw *p* = 0.0007, adjusted *p* = 0.990912).

*Homo sapiens* chromosome 20 open-reading frame 199 (C20orf199) has a significantly higher expression in exercise group compared to the non-exercise group after treatment (raw *p* = 0.0001, adjusted *p* = 0.990912).

The gene expression of SLC22A10 is significantly higher in exercise groups compared to the non-exercise group after treatment (raw *p* = 0.0002, adjusted *p* = 0.990911).

## Discussion

After a 3-month exercise intervention several genes showed higher expression compared to the control group. However, after adjusting for multiple testing there were no significant differences in expression observed. This is the first study investigating the chronic effects of resistance training on NK cell gene expression profile in breast cancer patients undergoing adjuvant therapy.

As mentioned in the section “Introduction,” the chronic effect of resistance exercise training on differential gene expression of NK cells has not yet been investigated. Dias et al. (2015) found that after 18 weeks of aerobic endurance training 211 gene transcripts involved in cell cycle regulation, proliferation, and development of immune cells were changed in PBMCs. A study conducted by Liu et al. (2017) identified 72 transcripts in PBMCs involved in encoding ribosomal proteins and oxidative phosphorylation. The study was conducted on young athletes compared to non-athletes with a false discovery rate <0.05 (Liu et al., 2017). However, one should keep in mind that PBMCs are a heterogeneous mix of immune cells. Detected changes in gene expression over time may also be driven by alterations of cell proportions.

Natural killer cells subsets make up with 90% as surface markers of CD56-dim, classified as cytotoxic and 10% as CD56-bright, classified as immune-regulatory and least mature (Timmons and Cieslak, 2008). Both these subsets are localized in different compartments and also differ in their expression of β_1_ and β_2_-adrenergic receptors with higher expression on CD56^dim^ NK cells and are therefore more responsive to exercise induced epinephrine (Bigley et al., 2012; Pedersen et al., 2016). In fact, Shephard (1995) has shown that exercise training leads to changes in immune cell subsets. Moreover, differences in results may also be explained by the different exercise regimes (aerobic vs. strength) as well as intensity and duration of exercises performed. The results of these studies do not provide evidence that chronic exercise, especially chronic resistance exercise impacts the transcriptomic profile of NK cells in breast cancer patients.

In contrast to chronic effects of exercise, acute exercise has been reported to induce chromatin remodeling in NK cells and changes the expression of NK cell receptors (Zimmer et al., 2015; Schenk and Zimmer, 2018). Zimmer et al. (2014) also showed that a single bout of exercise could regulate the cytokine levels which consequently affect the epigenome of NK cells. Crisafulli et al. (2013) conducted studies on chronic exercise effects did not show any change in NK cell cytotoxicity *in vitro* in post-menopausal overweight women compared to the stretching control group. Recent animal studies by Pedersen et al. (2016) have shown that NK cells were mobilized by epinephrine and were redistributed to tumors by IL6-dependent manner.

Few studies that have reported NK cell gene expression pattern, migration potential, cytotoxicity, and change in NK cell subset population are in context of acute physical exercise, data regarding chronic exercise are few and contradictory.

Our study showed that the exercise intervention had negligible effects on the gene expression. The transcript TET1 is responsible for DNA methylation and its expression seems to be slightly higher post exercise. The TET1 gene (10–11 translocation methyl cytosine dioxygenase 1) is an epigenetic marker responsible for the conversion of the modified DNA base 5-methylcytosine (5-mC) to 5-hydroxymethylcytosine (5-hmC) and is the initial step for active DNA demethylation in mammals. Reduced levels of TET1 have been associated with higher proliferation levels (Takai et al., 2014). The other interesting candidates were as follows: FXYD4, an ion transport regulator. VAPA, *Homo sapiens* VAMP-associated protein A has a role in RRAS signaling which in turn attenuates integrin beta-1 (ITGB1) activation at the cell surface (Weber-Boyvat et al., 2015). *Homo sapiens* WDR68 interacts with other enzymes and may play a significant role in a signaling pathway regulating cell proliferation (Gangula and Maddika, 2013). C20orf199, SLC22A10 is a solute carrier family protein. Apart from TET1, the above mentioned genes are well documented but may not have a relevant function in respect to NK cells function or cytotoxicity. Their function in NK cell still needs to be elucidated. The other transcripts with a significant *p*-value prior to adjustments for multiple testing are hypothetical predicted protein coding genes, and our literature search did not show any relevant known function. They neither interact with each other nor do they form a pathway pattern.

Physical exercise indeed disturbs the immune system homeostasis. However, our study together suggests that these changes are probably driven by acute rather than chronic exercise effects. Additionally, chronic exercise may not induce any changes in NK-cell gene expression in breast cancer patients undergoing medical treatment.

One of the possible explanations for our findings can be, since medical treatments (chemotherapy or radiation therapy) strongly influence gene expression in various tissues (Klebanoff et al., 2005); this can in turn affect the NK cell gene expression pattern and hence affect our findings. It is indeed a limitation in our study that we do not have healthy control group to compare our findings.

It is of importance to note that similar to other investigations in this field our study was limited by small number of participants and should thus be considered a pilot study. Another limitation is that we did not measure NK cell functions or the change in NK cell number or proportions. However, ours is the first study that explored effects of chronic resistance training program as well as cancer therapy effects on NK cell gene expression of breast cancer patients. Further research in this context should include acute and chronic assessments of NK cell function, transcriptomics, and epigenomics in larger sample sizes with different exercise modalities.

## Author Contributions

All authors listed have made a substantial, direct and intellectual contribution to the work, and approved it for publication.

## Conflict of Interest Statement

The authors declare that the research was conducted in the absence of any commercial or financial relationships that could be construed as a potential conflict of interest.
